# A sequential CO_2_ and 1540 nm laser for the treatment of neck skin laxity

**DOI:** 10.1111/srt.13469

**Published:** 2023-09-18

**Authors:** Stefania Belletti, Beatrice Marina Pennati, Francesca Madeddu

**Affiliations:** ^1^ Studio Kalos Milano Italy; ^2^ El.En. Group Calenzano Italy

Dear Editor,

Skin laxity is one of the most common aesthetic problems. There are several strategies adopted nowadays to improve it but the gold standard for skin resurfacing treatments remains the carbon dioxide (CO_2_) laser. Recent studies have demonstrated that treating the skin for remodelling with the CO2 wavelength (10 600 nm) and the wavelength 1540 nm together is very effective. This synergy seems to enhance the depth of stimulation and the shrinking impact.^1^, ^2^ Specifically, the consecutive action of CO_2_ and infrared wavelengths extends and intensifies the thermal effect, as shown by several published research studies. The fractionated emission modes ensure more successful therapies for tissue remodelling and guarantee a better healing process increasing cell turnover and stimulating further deep in the tissue.^3^, ^4^, ^5^, ^6^ For example, it is known that the 1540 nm wavelength in cultured fibroblasts promotes the expression of genes involved in collagen synthesis while suppressing the synthesis of matrix proteins.^7^, ^8^, ^9^, ^10^

The goal of this study was to evaluate the effectiveness of an innovative laser device (DuoGlide, DEKA M.E.L.A Srl, Florence, Italy) that combines the CO_2_ and 1540 nm wavelengths in the management of neck skin laxity using a recently developed scanning unit called μScan DOT. A precise balance between ablation and coagulation depths is possible because the scanner can deliver one or both wavelengths (1540 and 10 600 nm) in a sequential emission mode on the same DOT (columns of thermal damage). This is the area that interests both MAZs (Microscopic Ablation Zones) and MTZs (Microscopic Thermal Zones) in the tissue. This characteristic makes it possible to offer new and more effective treatments. For the treatment protocol we used, for the CO_2_ wavelength source was set Energy/DOT of 26 mJ, while for the 1540 nm source an Energy/DOT of 10 mJ. To increase safety during treatment, the scanner was equipped with a contact sensor. Clinical observations of notable improvements in wrinkles, acne scarring, photodamage, skin laxity and face rhytides have been reported in the literature.^11^

Moreover, research findings by Anna Kołodziejczak et al.^12^ showed that skin elastic properties were significantly altered by a series of non‐ablative fractional laser therapies, bipolar radiofrequency, or powerful pulse light treatments. They focused these procedures on the eye area to increase skin elasticity and lessen wrinkles, both in terms of their number and depth. Between them, the non‐ablative fractional laser therapy resulted in the greatest improvements. Its considerable effectiveness may be related to the fact that the applied laser therapy generally affects both the elasticity and the viscoelasticity of the skin. In general, numerous investigations have demonstrated that thermally treated tissues undergo dermis remodelling. These technologies' (non‐ablative fractional laser, RF, and IPL) purpose was to trigger the dermis' heat reaction. It has to do with the remodelling of connective tissue. Contraction of the ‘old’ collagen and stimulation of fibroblasts to make new fibres (elasticity) and proteoglycans (viscoelasticity) lead to an improvement in the tension and density of the skin. Moreover, an innovative study by Bonan et al.^13^ showed that the combination of the abovementioned wavelengths can be used in a surgical procedure like blepharoplasty.

We hereby report the results of the treatment for neck skin laxity with the combination of the abovementioned double wavelength sequential mode on two women of 72 (see Figure 1) and 73 (see Figure 2) years and Fitzpatrick phototype III. Patients underwent 3 sessions of treatment at 45‐day intervals. In addition, the days necessary for the expulsion of the fibrin plugs and the patient's downtime were monitored to evaluate skin recovery. The mean patient's downtime was 5 to 6 days and the average days required for expulsion of fibrin plugs were 5 to 6 days as well. Patients were asked to self‐evaluate the result of the treatment with a 4‐point Global Aesthetic Improvement Scale (GAIS) (None‐0, Slight‐1, Mild‐2, Excellent‐3) and all of them agreed on an ‘excellent’ outcome. Indeed, the mean difference between the perceived age by the patients after treatment and the real age was – 6/7 years. The Fitzpatrick Wrinkle Classification System (FWCS)^14^ was used by the physician to assess patients’ clinical improvement considering a scoring system where 0 means no wrinkles and 9 deep and numerous wrinkles. For the 72‐year‐old woman, it decreased from 8 before treatment to 4 after 3 months of follow‐up. For the other patient (73 years old), it went from 6 before treatment to 3 after 3 months of follow‐up. Side effects were monitored during and after the treatment and in both patients, mild erythema and oedema were present for 3–4 days and 24 h respectively post‐treatment.

**FIGURE 1:**
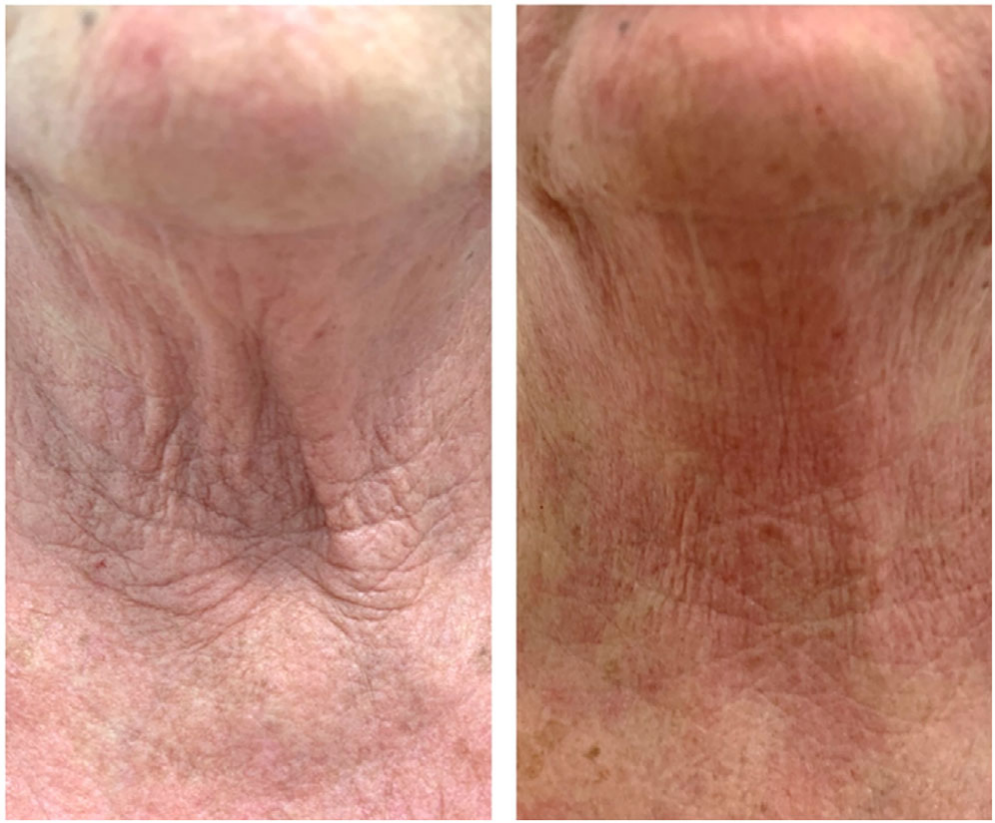
Sequential dual‐wavelength‐laser treatment for a 72‐year‐old woman patient. A comparison between the baseline (before treatment) and after 3 months of follow‐up is shown. She underwent 3 sessions at 45‐day intervals.

**FIGURE 2:**
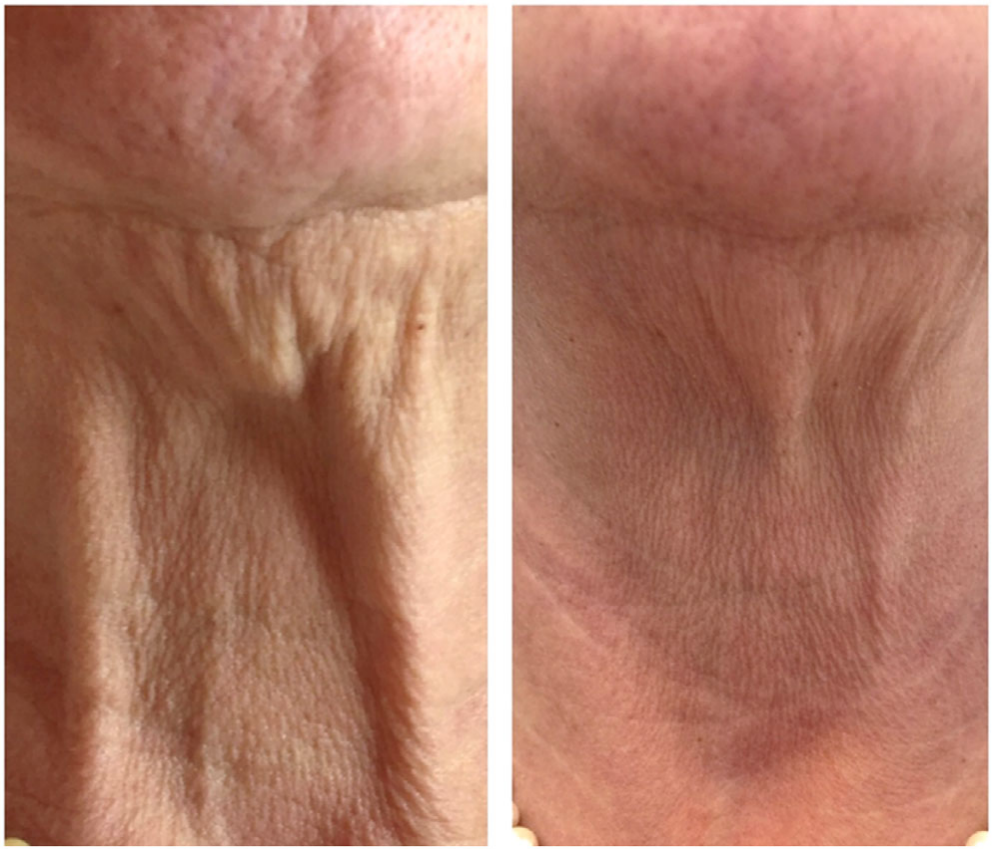
Sequential dual‐wavelength‐laser treatment for a 73‐year‐old woman patient. A comparison between the baseline (before treatment) and after 3 months of follow‐up is shown. She underwent 3 sessions at 45‐day intervals.

In conclusion, this sequential dual wavelength laser technology has the potential to be a new and safe non‐invasive solution for skin rejuvenation and the treatment of skin laxity in body areas very sensitive and difficult‐to‐treat. Additionally, with this cutting‐edge and effective procedure, it is feasible to get outcomes that are comparable to those seen with conventional CO_2_ laser resurfacing while significantly reducing the risk of scarring and hypopigmentation and speeding up the healing process. Due to their safety and expanding range of applications, these laser procedures are likely to guarantee that patients have an effective aesthetic treatment plan and results.

## CONFLICT OF INTEREST STATEMENT

BMP and FM are employed at El.En. Group. The other authors declare that the research was conducted in the absence of any commercial or financial relationships that could be construed as a potential conflict of interest.

## INSTITUTIONAL REVIEW BOARD STATEMENT

The study was conducted in accordance with the Declaration of Helsinki. As the device has been already CE‐marked device since 2021, ethical review and approval were waived for this study.

## INFORMED CONSENT STATEMENT

Informed consent was obtained from all subjects involved in the study.

## Data Availability

Data that support the study findings are available on request from the corresponding author.
